# First glimpse on attitudes of highly educated consumers towards cell-based meat and related issues in Brazil

**DOI:** 10.1371/journal.pone.0221129

**Published:** 2019-08-30

**Authors:** Júlia de Paula Soares Valente, Rodrigo Alonso Fiedler, Marina Sucha Heidemann, Carla Forte Maiolino Molento

**Affiliations:** Animal Welfare Laboratory, Federal University of Paraná, Curitiba, Paraná, Brazil; University of Florida, UNITED STATES

## Abstract

Our aim was to study Brazilian consumer attitudes towards cell-based meat and related issues. From 408 respondents from Curitiba and 218 from Joinville, the majority was women with higher level of education; 65.2% and 70.2% frequently consumed meat and 50.7% and 50.9% would not stop eating meat; 81.6% and 82.6% had little or no knowledge about cell-based meat. After watching an explanatory video, 41.9% and 34.4% stated they would eat cell-based meat without restrictions; 24.5% and 23.9% stated they would try depending on conditionals. Overall, 63.6% declared they would eat cell-based meat; among vegetarians and vegans, 24% and 8% stated they would eat cell-based meat, with additional 25.0% and 27.0% stating “it depends”; thus, the major public for cell-based meat seems to be meat eaters. Animal welfare was the principal reason for considering not eating meat and a major benefit of cell-based meat. In conclusion, the majority of respondents would not stop eating meat; additionally, they would eat cell-based meat.

## Introduction

In comparison to the last decades, global population growth up to 2050 is expected to be slower. Despite this fact, the Food and Agriculture Organization of the United Nations published a report showing that meat consumption tends to double midway through the century [1]. Higher consumption necessarily implies increases in meat production, with associated increases in environmental and animal ethics issues. Therefore, new substitutes for traditional animal protein are being researched [2]. Among the options is cellular agriculture, a new technology in food production that in the future may supply large amounts of high-quality protein [3]. Cell-based meat, also known by the names of clean, cultivated, synthetic, artificial, *in vitro*, lab-grown, cell-based and slaughter-free meat, is a novel and disruptive technology. The very name to this new meat is still undecided and a major issue, since it is known that the name influences how positive behavioral intentions towards the product will be [4].

In terms of methodology, cellular meat is based on muscular tissue culture starting with a sample of cells taken from a live animal [5, 6] and research continues to get this product increasingly similar to traditional meat in color, flavor, aroma, texture and palatability. Despite much progress in the field, there are technological barriers yet to be overcome for cell-based meat to be produced at large scale and become an alternative to conventionally produced meat [5]. One strategy comes from research with stem cells. In the last two decades, there has been great advance in the identification, selection and modification of stem cells in order to provide a series of viable cell types for *in vitro* meat cultivation [7]. However, some issues still require research before mass cell-based meat production can become effective [8], such as the development of a structure similar to the *in vivo* circulatory system, capable of providing sufficient nutrients and oxygen to allow for muscular tissue growth [5]. Regarding culture media, a key issue is the search for ingredients which are not from animal origin, such as alternatives to animal growth factors and blood serum [6]. Additionally, in muscle there are cells other than the specific differentiated muscle cells, which may be of value for new techniques for *in vitro* muscle cultivation. One example is the satellite muscle cells, which are derived from stem cells and are responsible for muscle regeneration after injury. Regarding myoblasts, there is not yet enough knowledge about how to maintain them at a constant state of multiplication and there might as well be subsets of such cells with even better regenerative ability [7]. Thus, there is room for much research, which is likely to produce better solutions than we can anticipate at this point in time.

A major advantage that favors the development of cell-based meat technology is lower environmental footprint as compared to the conventional system, estimated to be 7 to 45% less energy use, 78 to 96% less emission of greenhouse gases, 99% less land use, and 82 to 96% less water use [9]. However, some controversy exists regarding greenhouse gas emissions. A recent study conducted by Lynch and Pierrehumbert [10] compared greenhouse gas emissions in prospective scenarios for both conventional and cell-based meat production systems and reported that cultured meat results in less warming than cattle initially; however, in a scale of hundreds of years, cell-based meat may be more hazardous than the traditional method due to CO_2_ accumulation. Likewise, Mattick, Landis, Allenby and Genovese [11] proposed that, even though cell-based meat may require less agricultural inputs and land use, it might be associated to considerable energy consumption. Most importantly, due to the many uncertainties in terms of premises to be employed in these estimations, this area of study deserves continuing efforts.

It is yet another major advance of cell-based meat that especially motivates our work. In 2014 it was estimated that there were some 1.43 billion cattle, 1.87 billion sheep and goats, 0.98 billion pigs and 19.60 billion birds around the globe who were raised for the production of food and raw materials for the manufacture of products for human use [12]. Most of these billions of domestic animals live under abusive or neglect conditions of intensive farming [13]. Additionally, the anthropic biomass distribution to billions of domestic animals represents an intrinsic factor for the reduction of the number of wild animals sharing our planet, as humans and farm animals are biosequestering a major part of the total carbon atoms available in the planet [14]. It is recognized that, for example, the introduction of cell-cultured meat to the market presents the potential for reducing animal suffering caused by factory farming, because this alternative meat production system no longer requires the slaughter of animals [15]. However, the benefits for the animals may be much broader, as it will probably reduce both pressure for intensive farming of animals, with resulting benefits to domestic animal welfare, and the total number of domestic animals raised for food in the planet, with resulting benefits for the welfare of wild animals. Thus, the advantages of this innovation in the production of meat for animal welfare are indisputable.

For the benefits of cell-based meat to be attained, one important question is consumer acceptance. Hartmann and Siegrist [16] reviewed ten articles, and in eight of them, most consumers did not recognize the environmental impact caused by conventional meat production, whereas in the other two 58% and 64% of consumers stated such concerns. In their survey regarding the future consumption of cell-based meat, Verbeke, Marcu, Rutsaert, Gaspar, Seibt, Fletcher and Barnett [17] observed that 100% of the 179 participants were unaware of the subject and shocked by the possibility of *in vitro* multiplication of animal cells for human consumption. Another survey, which aimed to analyze consumer reaction to health problems that can arise due to consumption of traditional or cell-based meat, showed that people are more accepting of the risks offered by conventionally produced protein in comparison to protein produced through new technologies; however, it was emphasized that the amount of information available to participants on each production method was different, and such difference may have interfered in the results [18].

Additionally, it is likely that cultural characteristics play a role in the acceptance of cell-based meat and, thus, it seems relevant to understand the perception and opinions of consumers with different backgrounds. Most information available is based in data from European respondents, US and Australia [16] and advancements in knowledge from other geographical regions are warranted. The attitudes in countries which are major players in the meat industry scenario seem especially important to understand. In this sense, Brazil is recognized as second cattle, fourth pig and second broiler chicken producer in the world [19], raising approximately 215 million bovine, 41 million pigs and 1.42 billion broiler chickens [20, 21]. These numbers suggest that the attitudes of Brazilian consumers in relation to cell-based meat are central to the discussions, both because of their impact in domestic consumption and because Brazilian attitudes towards meat may influence the strategies the country will adopt in the future for its important meat exporting activities. Our goal was to advance knowledge on Brazilian consumer attitudes towards cell-based meat and related issues. Due to the Brazilian geographic and cultural diversity, this study aimed at an initial understanding of the attitudes of Southern Brazilians, by studying residents of the two biggest cities in the States of Paraná and Santa Catarina, Curitiba and Joinville, respectively.

## Materials and methods

Residents of Curitiba, most populous city in the State of Paraná and its capital, and Joinville, most populous city of the State of Santa Catarina, were invited to participate in an online survey through the Google Forms platform, from March to July 2018. The link to the survey was distributed via e-mail and published in social networks, in a snow-ball sampling method in which respondents were asked to further advertise the survey [22]. For both cities, the survey intended to reach the general public. Of a total of 739 respondents, 408 lived in Curitiba, 218 in Joinville and 113 in other cities; respondents from other cities were not considered in the analyses. Thus, responses from 626 participants were evaluated.

The minimum sample size for each city was calculated with the following formula [23]:

$$n=\frac{N*Z^{2}*p*(1-p)}{Z^{2}*p*(1-p)+e^{2}*(N-1)}$$

*n* = sample size; *N* = population size; *Z* = confidence level; *e* = margin of error; *p* = sample proportion.

According to the 2018 official estimate [24], population sizes were 1,917,185 people in Curitiba and 583,144 in Joinville, which resulted in sample sizes of 385 for Curitiba and 384 for Joinville (margin of error of 5% and level of confidence of 95%). Since total number of 218 respondents from Joinville was lower than calculated sample size for a margin of 5% error, the same formula was used to calculate error for a fixed sample of 218 respondents, resulting in a 6.6% margin of error. The convenience sampling method resulted in a sample that was characterized by an overrepresentation of highly educated respondents.

The questionnaire contained 14 questions, of either open-ended, multiple-choice or 5-point Likert scale types. Questions 1 to 6 asked demographic data, 7 to 11 approached opinions on conventional meat, and 12 to on cell-based meat. The short video *Clean Meat—A Vision of the Future* (https://www.youtube.com/watch?v=_GgP6jo5DTM&t=31s) was played with subtitles in Portuguese after question 12, to provide some level knowledge to all respondents and support their answers to questions 13 and 14. The video explains in simple terms the process used to obtain cell-based meat from a feather naturally shed by a broiler chicken.

Data were compared between cities and respondent gender, using either the Mann-Whitney test, for questions with more than two response options, or the chi-square test, for questions with only yes or no response options, which were performed with the software Minitab, version 18, with a level of confidence set at 95%.

This work was approved by the Ethics Committee on Research with Humans of the Federal University of Parana, protocol number 2501247 2018.

## Results and discussion

The majority of the respondents were women (69.6% in Curitiba and 58.3% in Joinville), aged between 20 and 29 (37.0% and 33.9%) years and with a university degree (59.6% and 66.1%, Table 1). The percentage of women in the general population is 52.3% in Curitiba and 49.6% in Joinville [24], thus lower than the percentage of women who participated in the study. The greater percentage of female respondents is probably related to low sampling control by the online survey [25]; there may also be a relevant effect of differences in interest between women and men in issues related to meat [26] and to animal welfare [27]. The majority of young respondents may be related to the internet and social media proximity and the awareness of animal welfare and ethics concerns by them [28, 29].

**Table 1:** Demographic data of 408 and 218 respondents from Curitiba and Joinville, respectively, in an online survey from March to July 2018.

Variable	Category	Number of respondents (%)		Total
		Curitiba	Joinville	
Gender	Female	284 (69.6)	127 (58.3)	411
	Male	124 (30.4)	91 (41.7)	215
Age (years)	≤ 19	56 (13.7)	19 (8.7)	75
	20–29	151 (37.0)	74 (33.9)	225
	30–39	78 (19.1)	50 (22.9)	128
	40–49	66 (16.2)	45 (20.7)	111
	≥ 50	57 (14.0)	30 (13.8)	87
Educational level	Up to high school	31 (7.6)	22 (10.1)	53
	University degree, incomplete	134 (32.8)	52 (23.9)	186
	University degree, complete	70 (17.2)	41 (18.8)	111
	Postgraduate degree	173 (42.4)	103 (47.2)	276

The low sampling control produced a major characteristic of our data set, the high percentage of respondents with university degrees, which is lower in the general population: 16.4% in Curitiba and 9.6% in Joinville [30]. This is most likely a result of the means used for participant invitation, through social network of authors and snowballing [22], which probably tended to involve more people within similar educational levels. Thus, our results represent mostly the perception and opinions of highly educated people and further work is needed to understand the views across educational groups, as well as other geographic regions in Brazil.

Regarding meat consumption, 85.3% of respondents from Curitiba and 89.4% from Joinville consumed it at least once a week (Table 2). Comparatively, in the only other paper on cell-based meat that reported data regarding frequency of meat consumption, Hoek, Luning, Weijzen, Engels, Kok & De Graaf [15] found that 89.7% of the British respondents and 94.0% of the Dutch consumed meat once to four times per week. In Curitiba, 14.7% of the respondents did not consume meat, and in Joinville, 10.6% (p = 0.28, Mann-Whitney). These percentages are in close range to the results of an official survey conducted by IBOPE [31], in which 14% of Brazilians described themselves as vegetarians. When consumption is analyzed according to gender, men showed significantly higher meat consumption compared to women (p < 0.01, Mann-Whitney). This result also seems compatible with the literature, as meat consumption is related to gender identity [32] and men are less enthusiastic to reduce meat consumption or shift to a plant-based diet [33].

**Table 2:** Frequency of meat consumption of 408 and 218 respondents from Curitiba and Joinville, respectively, in an online survey from March to July 2018, and of 253 British and 318 Dutch respondents as per Hoek et al., 2019 [15].

Respondent origin	Number of respondents	Meat consumption frequency Number of respondents (%)		
		No consumption	Some consumption: 1 to 3 (C and J) or 1 to 4 (UK and N) times a week	High consumption: 4 (C and J) or 5 (UK and N) times a week or more
Curitiba (C)	408	60 (14.7)	42 (19.3)	266 (65.2)
Joinville (J)	218	23 (10.5)	82 (20.1)	153 (70.2)
United Kingdom (UK)	253	26 (10.3)	156 (61.5)	71 (28.2)
Netherlands (N)	318	19 (6.0)	132 (41.5)	167 (52.5)

On the importance of meat, 56.4% of respondents from Curitiba and 56.9% of those from Joinville stated that meat is important in their diet (p = 0.99, Mann-Whitney). The consumption of meat was reported as very important by 42.7% of men and 28.5% of women (p < 0.01, Mann-Whitney). The results are similar to those by Schösler, de Boer, Boersema and Aiking [34], who reported high association between masculinity and meat consumption in Turkey. The percentage of respondents who stated that meat is important in their diet is lower than that of those who declared to be meat consumers, which may reveal some flexibility towards vegetarian diets. Such flexibility may be associated with the rapid growth of vegetarianism in many countries, as is the case in Brazil. In official surveys conducted by IBOPE [31], 8% of Brazilians declared to be vegetarians in 2012 and 14% in 2018 [35], demonstrating an increase of 75% over a period of six years.

To the open-ended question about the perception of any problem with the consumption of meat, 48.2% of the people from Curitiba and 55.1% from Joinville answered that they perceived no problem (p = 0.10, Mann-Whitney). Among those who stated that they perceived a problem, the main themes reported were similar in both cities: (1) animal welfare (69.4% Curitiba and 48.1% Joinville, 62.9% overall for those who stated that they did see a problem), reporting words such as cruelty, suffering and slaughter; (2) environmental issues (36.1% Curitiba and 46.9% Joinville, 39.4% overall), with words such as pollution, deforestation and water use; and (3) human health (27.9% Curitiba and 27.2% Joinville, 27.7% overall), citing cancer and digestive problems, amongst other health issues. An interesting result which deserves further research is the evident top priority for meat problems related to animal welfare, cited by almost the double number of respondents (62.9%) as compared to the second theme, environmental issues (39.4%). The same gender pattern observed for other answers appeared again, with a greater proportion of men (65.6%) reporting no problem in meat consumption in comparison to women (49.4%) (p < 0.01, Mann-Whitney).

Table 3 shows the percentages of yes and no answers to the question on whether the respondent would stop eating meat. In both cities, answers concentrated around 50% for yes and no (p = 0.97, chi-square), with an overall average of 49.2% for yes and 50.8% for no. The main reasons given by those who answered yes were animal welfare (49.2% Curitiba and 36.1% Joinville, 44.9% overall for those who responded yes), human health (25.9% Curitiba and 32.0% Joinville, 27.9% overall) and the environment (17.8% Curitiba and 27.8% Joinville, 21.1% overall). These results show once more animal welfare as the most cited argument. Regarding the willingness to stop eating meat, the majority of women (58.6%) would stop eating meat, while the majority of men (68.8%) would not stop eating meat (p < 0.01, chi-square).

**Table 3:** Answers to the question “Would you stop eating meat?” given by 408 and 218 respondents from Curitiba and Joinville, respectively, in an online survey from March to July 2018.

Variable	Category	Would you stop eating meat? Number of respondents (%)		P-value
		Yes	No	
City	Curitiba	201 (49.3)	207 (50.7)	0.97
	Joinville	107 (49.1)	111 (50.9)	
Gender	Female	241 (58.6)	170 (41.4)	< 0.01
	Male	67 (31.2)	148 (68.8)	

Regarding alternatives to meat, 70.9% of respondents knew at least one substitute (71.6% in Curitiba and 69.7% in Joinville; p = 0.63, chi-square and 75.2% of women and 62.8% of men; p < 0.01, chi-square). The main substitutes mentioned were grains (70.9% Curitiba and 77.6% Joinville, 73.2% overall for respondents who stated that they knew meat substitutes), vegetables in general (32.9% Curitiba and 30.3% Joinville, 32.0% overall) and eggs (11.6% Curitiba and 11.2% Joinville, 11.5% overall). According to Day [36], a plant-based diet is nutritionally adequate to human health and its greatest sources of protein are cereal grains and food legumes, as long as the average individual consumption of protein for maintenance remains 0.8 grams per kilogram, as following the recommendation of The Academy of Nutrition and Dietetics [37]. In addition, Parker and Valdiveloo [38] reported that there is no consensus over health outcomes in consuming eggs but whole grains remain associated with defense regarding some diseases. Hence, our results suggest some knowledge of respondents over meatless diets.

Regarding the specific knowledge on cellular agriculture, 81.6% of respondents from Curitiba and 82.6% from Joinville said they had little or no knowledge of the subject (p = 0.87, Mann-Whitney). Comparing gender, 82.7% of women and 80.5% of men knew little or nothing on the subject (p = 0.36, Mann-Whitney). Verbeke, Sans and Van Loo [39] reported that 51% of Belgium respondents were completely unaware of cellular agriculture, while 36% had heard about it but did not know what it meant. Similarly, our results show that a small percentage of respondents presented knowledge on cell-based meat. Additionally, this finding is likely influenced by the fact that our respondents held higher education degrees as compared to the average Brazilian population that may present even lower levels of knowledge regarding cell-based meat. However, it is our perception that this level of knowledge is embedded in a dynamic pool of information, and as such it is likely to rapidly improve, since news regarding cell-based meat is increasingly more frequent in different types of media.

After watching the video, respondents were asked if they would eat meat from cellular agriculture. Many respondents (41.9% in Curitiba and 34.4% in Joinville, 39.3% overall, p < 0.03, Mann-Whitney) answered positively without any conditionals (Table 4). The significant difference between cities reiterates the important influence of geographical and cultural aspects and warrants further studies. Since both cities are located in the South Region of Brazil, 130 km apart, probably larger differences will be observed amongst other Brazilian geographical regions or across different countries. In addition, respondents answered “I do not know” (20.6% Curitiba and 23.9% Joinville, 21.7% overall) or “it depends” (24.5% Curitiba and 23.9% Joinville, 24.3% overall) on factors such as taste, health consequences, price, and further information about the subject. Thus, the potential acceptance for cell-based meat in Brazil seems high, with sufficient room for this new technology to be part of the meat market.

**Table 4:** Answers to the question “Would you eat meat originated from cellular agriculture?” given by 408 and 218 respondents from Curitiba and Joinville, respectively, in an online survey conducted from March to July 2018.

Variable	Category	Would you eat meat originated from cellular agriculture? (%)				P-value
		Yes	It depends	I do not know	No	
City	Curitiba	171 (41.9)	100 (24.5)	84 (20.6)	53 (13.0)	0.03
	Joinville	75 (34.4)	52 (23.9)	52 (23.9)	39 (17.9)	
Gender	Female	162 (39.4)	89 (21.7)	104 (25.3)	56 (13.6)	0.68
	Male	84 (39.1)	63 (29.3)	32 (14.9)	36 (16.7)	

In 2015, Verbeke et al. [17] conducted a study with Belgian, Portuguese and UK citizens divided into an online and focus groups; both were presented to risks and benefits of red meat. It was observed that 66.7% of them would probably try cell-based meat, while 23.9% would certainly consume it. The difference in results between Verbeke et al. [39] and this study may be influenced by the different methods used to apply the questionnaires. Likewise, two other studies employing online surveys presented to respondents information about advantages of cultured meat to environment and human health: Gasteratos and Sherman [40] surveyed 3,219 students from Florida Atlantic University (U.S.), 1,538 U.S. citizens and 314 Australian citizens, and Mancini and Antonioli [41] applied a questionnaire to 525 Italian citizens. Florida students stated they would probably consume cell-based meat (34%), and 39% would definitely consume it, followed by 20% of the US citizens who would probably and 43% who would definitely consume it. Regarding Australian citizens, 36% would probably and 25% would definitely consume it. In relation to the Italian population, 54.5% were willing to try cultured meat, while 24.4% answered “maybe”; it is relevant to emphasize the sample was not representative of Italian population, since respondents were significantly younger and better educated than overall population, similar to the present study. Thus, considering current knowledge there is a trend for a relevant proportion of consumers to try cell-based meat.

The scenario seems different for the responses from vegetarian and vegan participants. Amongst them, only 23.3% (14 of 60 respondents) and 17.4% (4/23) stated that they would eat meat from cellular agriculture, with additional 20.0% (12/60) and 8.7% (2/23) stating “it depends”. Thus, results suggest that cell-based meat is likely to be consumed especially by those who include meat in their diets, which was reported also by Wilks and Phillips [42] and Bryant et al. [4]. However, it is important to note that percentages of non-meat eaters who stated their interest in trying cell-based meat are not negligible.

Considering the size and importance of the global meat industry, even a small percentage of the population willing to eat meat from cellular agriculture represents a relevant market. However, our work adds to the available information on consumer acceptance of cell-based meat, suggesting that in Brazil the percentage of consumers who accept this innovation seems to be relevant. This also seems to be the understanding of major players in the world economy, as suggested by the financial input of large investors, noticeably investors outside the field of food production, in start-ups that aim to scale cellular agriculture up to industrial level [43]. Additionally, results suggest the importance of studying the consequences of a partial shift in meat sourcing, from animals to cells, to mitigate negative and enhance positive effects of such a change, in all its dimensions, such as animal ethics, environmental, social, governance and economic aspects.

**Fig 1:**
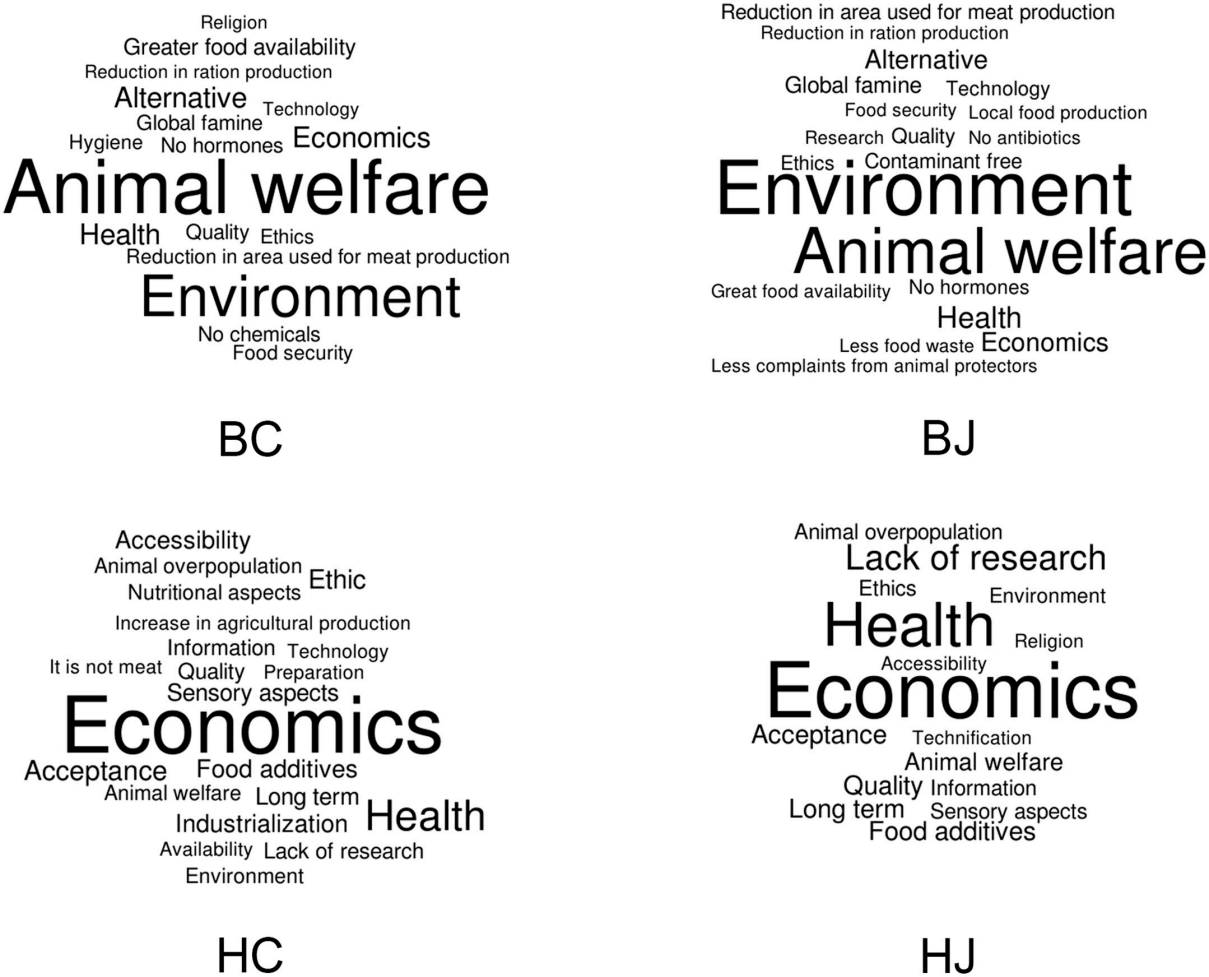
Benefits and harms of cell-based meat according to respondents from Curitiba and Joinville. (BC) Benefits indicated by 408 respondents from Curitiba; (BJ) Benefits indicated by 218 respondents from Joinville; (HC) Harms indicated by 408 respondents from Curitiba; (HJ) Harms indicated by 218 respondents from Joinville.

The last question of the survey was on the potential benefits and harms of introducing cell-based products to the market. The answers were categorized by keywords which were used to create the word clouds presented in Fig 1. In Curitiba, the most mentioned benefits were animal welfare (49.5%), environment (33.8%) and food alternatives (9.3%), whereas in Joinville they were environment (37.6%), animal welfare (30.7%) and health (7.8%). The most quoted harms, on the other hand, were economic (35.5%), health (13.7%) and ethics (3.9%) according to respondents from Curitiba, and economic (28.0%), health (19.7%) and lack of research (7.8%) according to respondents from Joinville. Furthermore, 14.0% of respondents from Curitiba and 18.3% from Joinville did not know or did not comment on benefits, whereas 25.2% from Curitiba and 32.6% from Joinville did not know or did not comment on harms. Lastly, 8.3% respondents from Curitiba and 2.8% from Joinville stated that there was no harm. It is interesting to highlight the use of the word “health” for both benefits and harms. This may be due to the perceived relation of health issues to meat consumption [41], as well as to unnaturalness [44] and fear of the unknown [39]. This finding warrants further research, to better understand the details people associate to both benefits and harms when they think of human health.

A major outcome in our word clouds (Fig 1) is the term animal welfare. Wilks and Phillips [42] also outlined animal welfare improvement as one of the general perceptions of respondents in their study, followed by the awareness of reduction in global warming; however, their study presented as major barriers to cell-based meat some ethical concerns and price. Laestadius and Caldwell [44] also noted major public comments in relation to benefits to animal welfare, environment and human health, with the perception of economic challenges related to high prices of cultured meat.

## Conclusion

This is the first report on public attitudes towards cell-based meat in Brazil. Awareness of cell-based meat by highly educated Southern Brazilians was low; however, after watching an explanatory video more than half of respondents stated that they would consume it, even though part of them included some conditionals such as taste, health consequences, price, and more information about the subject. Men stated consuming more meat, giving it more importance and seeing fewer problems with conventional meat consumption as compared to women, who in turn are more willing to try cell-based meat. A greater proportion of meat-eaters are willing to try cell-based meat as compared to vegetarian and vegan respondents; nevertheless, the percentage of non-meat eaters willing to try cell-based meat is not negligible. Overall, the potential acceptance for cell-based meat in Brazil seems high, with sufficient room for this new technology to be part of the meat market. Animal welfare appears as the principal reason given by respondents who would stop eating meat, as well as a major benefit of cell-based meat; other concerns stated by respondents were related to environment and health. Considering acceptance by the public, cellular agriculture seems to have potential to become more than niche market, reaching a considerable number of consumers. More research is needed to understand opinions in groups with lower educational levels as well as those from other geographical regions. Studies to better understand the underlying reasons for the concerns commonly observed in scientific literature regarding traditional and cell-based meat are also warranted.

## Supporting information

S1 Table Full dataset with date and time of respondent participation and all answers from Curitiba and Joinville respondents related to cell-based meat. (XLSX)
